# LncRNA SNHG3 enhances BMI1 mRNA stability by binding and regulating c‐MYC: Implications for the carcinogenic role of SNHG3 in bladder cancer

**DOI:** 10.1002/cam4.5316

**Published:** 2022-10-08

**Authors:** Jinbo Xie, Jinliang Ni, Huajuan Shi, Keyi Wang, Xiaoying Ma, Wei Li, Bo Peng

**Affiliations:** ^1^ Department of Urology, Putuo People's Hospital, School of Medicine Tongji University Shanghai China; ^2^ Department of Urology, Shanghai Tenth People's Hospital, School of Medicine Tongji University Shanghai China; ^3^ Department of Laboratory Medicine and Pathobiology University of Toronto Toronto Ontario Canada; ^4^ Department of Laboratory Medicine, Keenan Research Centre for Biomedical Science St. Michael's Hospital Toronto Ontario Canada; ^5^ Shanghai Clinical College Anhui Medical University Shanghai China

**Keywords:** bladder cancer, BMI1, c‐MYC, noncoding RNA, SNHG3

## Abstract

The transformation of nonmuscle‐invasive bladder cancer (BLCa) to muscle‐invasive type and distant metastasis are the two major threats to patients after surgery. Thus, it is important to identify the key genes of BLCa cell invasion and metastasis. Long noncoding RNA (lncRNA) is a potential clinical tool for cancer diagnosis and treatment. Herein, we verified that lncRNA SNHG3 is upregulated in human BLCa specimens and is proportional to poor clinical prognosis via a combination of bioinformatic analyses and wet bench experiments. Then, we constructed SNHG3 knockdown and overexpression cell models via lentiviral packaging and CRISPR‐Cas9 technique. Fluorescence in situ hybridization assay showed that SNHG3 is distributed in both the nucleus and cytoplasm of BLCa cell lines. In vitro assays including CCK‐8, EdU, colony formation, wound healing, transwell, and tube formation demonstrated that SNHG3 knockdown and overexpression potently inhibited and enhanced BLCa cell proliferation, migration, invasion, and angiogenesis. In addition, IVIS imaging revealed that SNHG3 knockdown could significantly inhibit M‐NSG mice xenograft tumor growth. Next, RNA sequencing, bioinformatics analyses and western blots indicated that SNHG3 could promote c‐MYC expression. RNA immunoprecipitation, actinomycin D assay and western blot assays suggested that SNHG3 could also bind c‐MYC protein which subsequently facilitate the stabilization of BMI1 mRNA, thus enhancing BMI1 protein level. However, SNHG3 knockdown had a slightly weaker inhibitory effect on BMI1 expression than c‐MYC knockdown. Further, in vitro assays demonstrated that BMI1 knockdown could suppress the SNHG3 activation‐induced tumor promoting effect in BLCa cells. Overall, this study has provided new insights into the potential implication of lncRNA SNHG3 in the pathogenesis of BLCa. Importantly, SNHG3/c‐MYC/BMI1 axis may be a novel target for regulating tumor growth and metastasis in BLCa patients.

## Graphical abstract

The schematic diagram illustrated the molecular mechanism that lncRNA SNHG3 plays an oncogenic role in BLCa: SNHG3 can stabilize BMI1 mRNA by binding to c‐MYC protein or regulating the expression of c‐MYC.

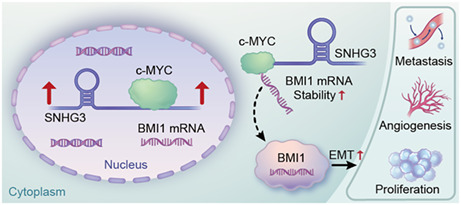

## 1 INTRODUCTION

Within the past few decades, the development of clinical treatment strategies for bladder cancer (BLCa) has encountered a bottleneck, mainly reflected in the prone to recurrence, the transition of BLCa from nonmuscle invasive (NMIBC) to muscle invasive (MIBC) and distant metastasis.^1^ Using bioinformatics, high‐throughput sequencing and molecular biology technologies to reveal the molecular mechanism of BLCa tumorigenesis and progression will provide a theoretical basis for the development and validation of therapeutic targets.

Long noncoding RNAs (lncRNAs) with more than 200 nucleotides are involved in a variety of biological processes, the roles of which in malignancies has been reported previously, and which can act as an oncogene or tumor suppressor gene.^2^, ^3^ Zhai W et al.^4^ demonstrated that lncRNA URRCC promotes renal carcinogenesis through the EGFL7/P‐AKT/FOXO3 signaling axis. In addition, Gong et al.^5^ demonstrated that lncRNA MEG3 inhibits renal cell carcinoma progression through the c‐Jun/ST3Gal1/EGFR axis. It has been reported that the regulatory effect of lncRNA on genes depends on its subcellular localization.^6^ When lncRNA is mainly localized in the cytoplasm, it can competitively bind microRNA (miRNA), thereby alleviating the transcriptional repression effect of miRNA on target genes. Jiang L et al.^7^ demonstrated that the lncRNA HOXA‐AS2 promoted the expression of HOXA3 by competitively binding to miR‐15a‐5p, thereby promoting the progression of thyroid cancer. The above‐mentioned lncRNA/miRNA/mRNA axis is termed as the competing endogenous RNA (ceRNA) mechanism, which has been widely reported in various cancers including BLCa.^8^, ^9^ However, most of the lncRNAs localize within in the nucleus and cytoplasm. Also, there are some lncRNAs mainly distributed within the nucleus. Obviously, it is impossible to elucidate all biological regulatory effects of lncRNAs using the ceRNA mechanism. Wei Liu et al.^10^ discovered a lncRNA Malat1 distributed only in the nucleus of mouse RAW264.7 cells, which regulates the activation of TDP43 through RNA‐binding protein (RBP), thereby affecting innate responses and autoimmune pathogenesis. Another study revealed an important regulatory role of splicing RBP in the subcellular localization of lncRNA by bioinformatics.^11^ Therefore, exploring the direct interactions between lncRNAs and intracellular proteins can help complement the regulatory network of lncRNAs in tumors.

To begin this work, we analyzed multiple RNA‐sequencing data sets and screened out two lncRNAs including SNHG1 and SNHG3. The small nucleolar RNA host genes (SNHGs) are a family of transcripts containing more than 20 members,^12^ some of which have been shown to be present in tumor tissues, including nonsmall‐cell lung cancer,^13^ acute myeloid leukemia,^14^ and urologic cancers.^12^ Several research groups have explored and elucidated the role of the SNHGs in cancer initiation and progression.^15^, ^16^, ^17^, ^18^, ^19^ It is worth noting that the regulatory role of the SNHG family in BLCa proved to be quite complicated.^12^ Our previous study demonstrated that SNHG1 can exacerbate the malignant behavior of BLCa cells through the miR‐493‐5p/ATG14/autophagy pathway.^20^ It seems SNHG3, our candidate lncRNA, has attracted more attention from different groups, which implies that SNHG3 is a biomarker with potential clinical translation value. In 2020, Dai G et al.^21^ demonstrated that SNHG3 promotes BLCa cell proliferation and metastasis through miR‐515‐5p/GINS2 axis. We initiated this work in 2019, and we verified that SNHG3 distributes both in cytoplasm and nucleus of BLCa cells by two different assays including fluorescence in situ hybridization and subcellular fraction real‐time PCR assays. Compared to the work of Dai G et al.,^21^ we more deeply explored the biological function of SNHG3 in the nucleus. In 2021, we noticed Cao Y et al.^22^ also investigated the regulatory effects of SNHG3 on BLCa progression. However, the limitation is that it is only validated in vitro, and the molecular mechanism has not been fully elucidated. Therefore, it is of great significance to continue to deeply investigate the role of SNHG3 in BLCa. Based on the pre‐experimental results, we hypothesized that SNHG3 can directly bind to target proteins or DNA to regulate the biological behavior of BLCa cells.

## 2 METHODS

### 2.1 Bioinformatics analysis

Gene expression matrices of BLCa samples were obtained from The Cancer Genome Atlas (TCGA) database (genome‐cancer.ucsc.edu/) and the Gene Expression Omnibus database (http://www.ncbi.nlm.nih.gov/geo/). We run the software R 4.0.2. The data were preprocessed with the dplyr package, differentially expressed genes were screened with the DESeq2 package, heatmaps were generated with the Complex Heatmap package, and data intersections were visualized with the Venn Diagram package.

### 2.2 Patients and tissues

A total of 58 pairs of BLCa and adjacent normal tissues were collected from January 2011 to December 2015 in Shanghai Tenth Hospital, School of Medicine, Tongji University. All the patients were histologically diagnosed with bladder urothelial cell carcinoma, none of which had received any radiotherapy or chemotherapy before surgery. In addition, all the surgeries including transurethral resection of bladder tumor and radical cystectomy were performed by the same surgical team. The clinical information details of patients are respectively shown in Supplemental Section 3.1 and 3.2. This work was approved by the Ethics Committee of Shanghai Tenth People's Hospital (Lot number SHSY‐IEC‐4.1/19–209/01), and all participants provided written informed consents.

### 2.3 Transfection

To knock down BMI1 and c‐MYC, 5637 and T24 cells were transfected with small interfering RNAs purchased from (IBSBIO). Cells were seeded in 6‐well plates and incubated to 50% confluence. Then, 50 nM siRNA (siBMI1 and sic‐MYC), which targeted BMI1 and c‐MYC, as well as 50 nM negative control siRNA (siCTRL) were transfected using the Lipofectamine® 3000 reagent (Invitrogen, USA). After transfection, cells were incubated for 24 h at 37°C. To overexpress SNHG3, 5637 cells were transfected with pcDNA3.1(+)‐SNHG3 and incubated at 37°C for 48 h.

### 2.4 Construction of stable BLCa cell lines

To stably silence SHNG3 gene expressions in BLCa cell lines, single guide RNAs (sgRNA) targeting SNHG3 and negative control (CTRL sgRNA) were designed and synthesized by ZONRIN (ZONRIN Biological Technology Co., LTD, China). T24 and J82 cells were infected with lenti‐cas9, then, puromycin was used to screen the infected cells. The full‐length sequence of SNHG3 and sgRNA sequences targeting SNHG3 are shown in Supplemental Sections 4 and 5.

### 2.5 Tube formation assay

The tube formation assay was performed to evaluate in vitro angiogenesis activities of different transfected cells. Briefly, 300 μl Matrigel (BD Biosciences) was plated into 24‐well‐plates and incubated at 37°C for 2 h to solidify. Then, human umbilical vein endothelial cells (HUVECs) were cultured on top of the Matrigel with different transfected cell supernatants containing cellular secretions. After incubation for 3 h at 37°C, tubes were observed under microscope and evaluated using Image‐Pro Plus 6.0 software to measure cumulative tube numbers.

### 2.6 Fluorescence in situ hybridization (FISH) assay

The specific probe for SHNG3 was hybridized with transfected cells for site hybridization. Assays were performed by the FISH kit (RiboBio, China), according to the manufacturer's protocol. The T24 and J82 cells were stained with 4, 6‐diamidino‐2‐phenylindole (DAPI; Beyotime), washed thrice using PBS, and imaged by microscopy (Olympus BX53 Biological Microscope).

### 2.7 H&E staining

Tissues to be stained were fixed on slides. First, sections were dried at 60°C, deparaffinized with xylene, dehydrated with gradient ethanol, and stained with eosin and hematoxylin, successively. Finally, slides were sealed with neutral gum, and imaged using an optical microscope.

### 2.8 Immunohistochemical (IHC) staining

Tissues were embedded in paraffin, then sectioned into 4 μm thick slides, deparaffinized in xylene, rehydrated in a graded series of alcohol, and blocked with goat serum. After being stained with primary antibodies at 4°C overnight, sections were washed thrice then incubated with the secondary antibody for 20 min. After that, sections were reacted with 3,3′‐diaminobenzidine and counterstained with hematoxylin. Images were obtained by microscopy. Normal and tumor tissues were scored by evaluating their staining percentages. All the antibodies were listed as follows: anti‐Ki67 (ab15580, Abcam), anti‐E‐cadherin (ab231303, Abcam), anti‐SLUG (ab128485, Abcam), anti‐c‐MYC (ab32072, Abcam), anti‐BMI1 (ab126783, Abcam), and Goat anti‐Rabbit IgG H&L (ab205718, Abcam).

### 2.9 RNA sequencing analysis

We extracted total RNA from SNHG3‐knockdown T24 cells and control T24 cells and prepared three parallel samples for each group. The samples were sent to Personal Biotechnology for purification and sequencing. We run software R4.0.2. Data were preprocessed with the dplyr package, differentially expressed genes were screened with the DESeq2 package, heatmaps were generated with the Complex Heatmap package, and volcano maps were generated with the Enhanced Volcano package. The gene set enrichment analysis (GSEA) was used for Hallmark and KEGG analysis.

### 2.10 RNA immunoprecipitation (RIP) assay

To evaluate the binding affinity between c‐MYC and the RNAs (SNHG3 and BMI1), The RIP assay was performed using the EZ Magna RIP Kit (Millipore). Briefly, total RNA was isolated from transfected cells using the TRIzol reagent (Invitrogen). Then, incubation with isolated and purified RNA was performed with buffer containing magnetic beads with anti‐c‐MYC antibody (ab32072, Abcam) or Rabbit IgG isotype control (2729, CST, USA). The immunoprecipitated RNA isolated by RIP was analyzed using qRT‐PCR, and the primers were listed in Supplemental Section 6.

### 2.11 In vivo xenograft tumor model

Tongji University Laboratory Animal Welfare Ethics Review working Group Committee approved the use of animals in this study. NOD‐Prkdc^scid^ Il2rg^em1^/Smoc mice (M‐NSG)^23^ (Male, 4 weeks) were purchased from the Shanghai Model Organisms Center and used to develop cell derived xenograft models. The stable transfected T24 cells were prepared as cell suspensions and injected into the right axilla of M‐NSG mice to establish xenograft models including three groups: CRTL sgRNA, SNHG3 sgRNA2, and SNHG3 sgRNA3. After inoculation, tumor lengths and widths were measured and recorded. IVIS was used to assess tumor volumes during feeding.

### 2.12 Statistical analysis

The SPSS software 24.0 and GraphPad Prism 8.0 were used for data analyses. Normally distributed continuous data are presented as means ± SD. A two‐sided Student's *t*‐test was used to evaluate between group differences; a Chi‐square test was used to analyze associations between SNHG3 expressions and clinicopathological features, while the Kaplan–Meier method and log‐rank test were used for survival analyses. *p* < 0.05 was considered statistically significant.

## 3 RESULTS

### 3.1 SNHG3 is upregulated in human BLCa specimens and positively correlated with poor prognosis

**FIGURE 1:**
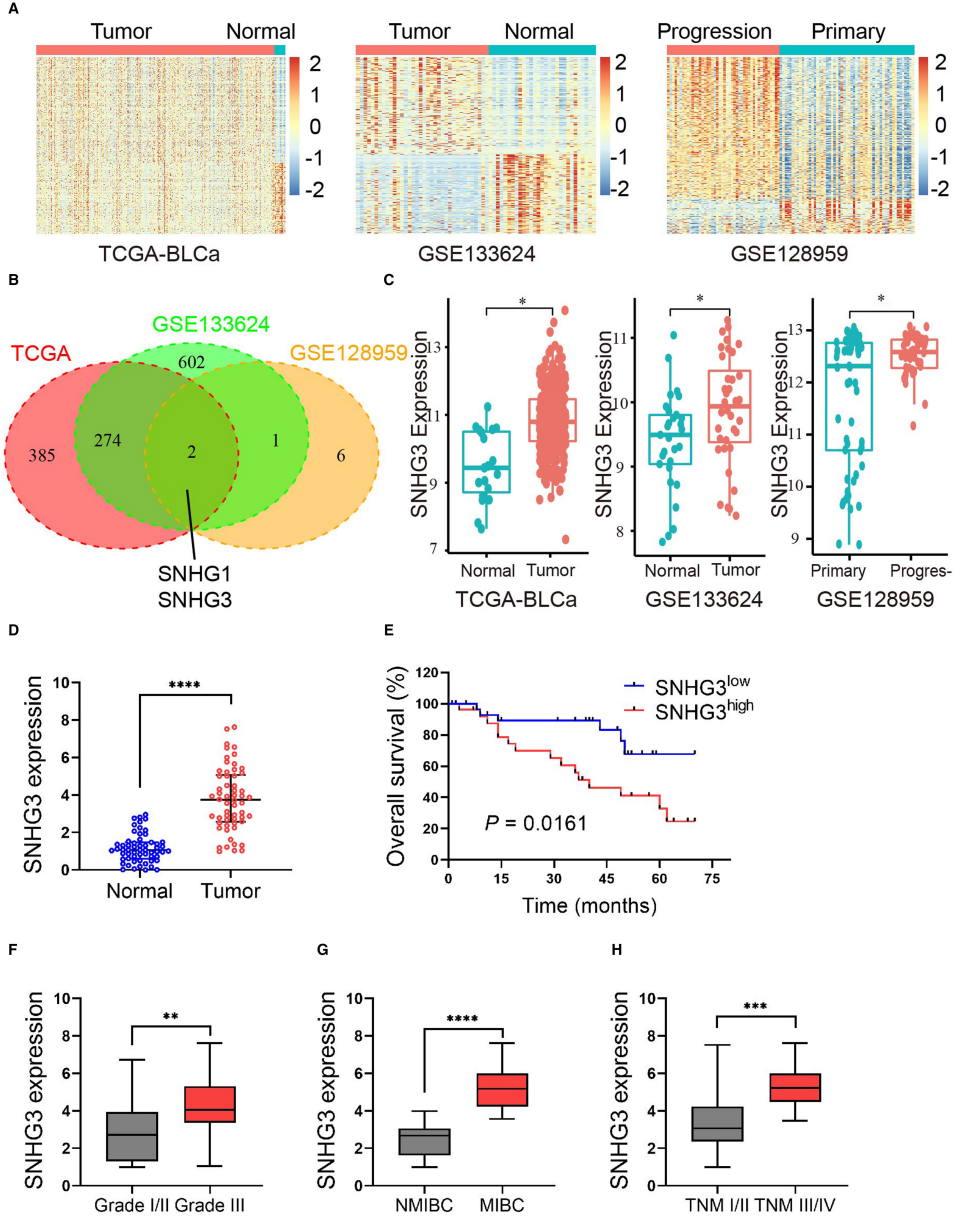
SNHG3 is upregulated in human BLCa specimens and positively correlated with poor prognosis. (A). The heatmap of the gene expression profile of BLCa data set, GSE133624 and GSE128959 downloaded from TCGA and GEO Database. (B) The results of these three data sets were intersected to obtain 24 abnormally overexpressed RNA transcripts in BLCa. Of these, only SNHG1 and SNHG3 belonged to lncRNA. Adjusted *p* value<0.05, log2FC >0.5 was used as screening criteria to identify differentially expressed RNAs. (C) Relative SNHG3 expression in BLCa tissues and paracancerous tissues and the data were collected from BLCa data set—TCGA, GSE133624‐GEO, and GSE128959‐GEO. (D) qRT‐PCR results showed that the expression of SNHG3 was significantly higher in BLCa tumor tissues compared with paracancerous tissues (58 cases of paired paracancerous and cancer tissues). (E) Kaplan–Meier analysis showed that the expression level of SNHG3 was proportional to the poorer overall survival of BLCa patients (*p* = 0.0161). (F–H) qRT‐PCR results showed that the expression level of SNHG3 was significantly correlated with clinicopathological parameters, including tumor grade, myometrial invasive, and TNM staging. **p < 0.05, **p < 0.01, ***p < 0.001, ****p < 0.0001.

First, we extracted the BLCa data set from TCGA and GEO (GSE133624, GSE128959) data sets, respectively, and then screened the differentially expressed genes from the raw matrix file (Figure 1A). The intersection of the results of these three data sets revealed 24 RNA transcripts aberrantly overexpressed in BLCa (Supplemental Section 2), of which only SNHG1 and SNHG3 belonged to lncRNAs **(**Figure 1B **)**. Both bioinformatics analysis and qRT‐PCR assays showed that SNHG3 was significantly more expressed in BLCa tumor tissues compared with paracancerous tissues (58 cases of clinical paired paracancerous and cancer tissues; Figure 1C, D). Kaplan–Meier analysis suggested that the expression level of SNHG3 was proportional to poorer overall survival in BLCa patients (Figure 1E). In addition, our clinical data combined with qRT‐PCR results demonstrated that the expression level of SNHG3 was significantly correlated with clinicopathological parameters, including tumor grade, muscle invasion, and TNM stage (Figure 1F–H; Supplemental Sections 3.1 and 3.2).

### 3.2 Knockdown of SNHG3 inhibits BLCa cell proliferation, invasion, migration, and angiogenesis in vitro

**FIGURE 2:**
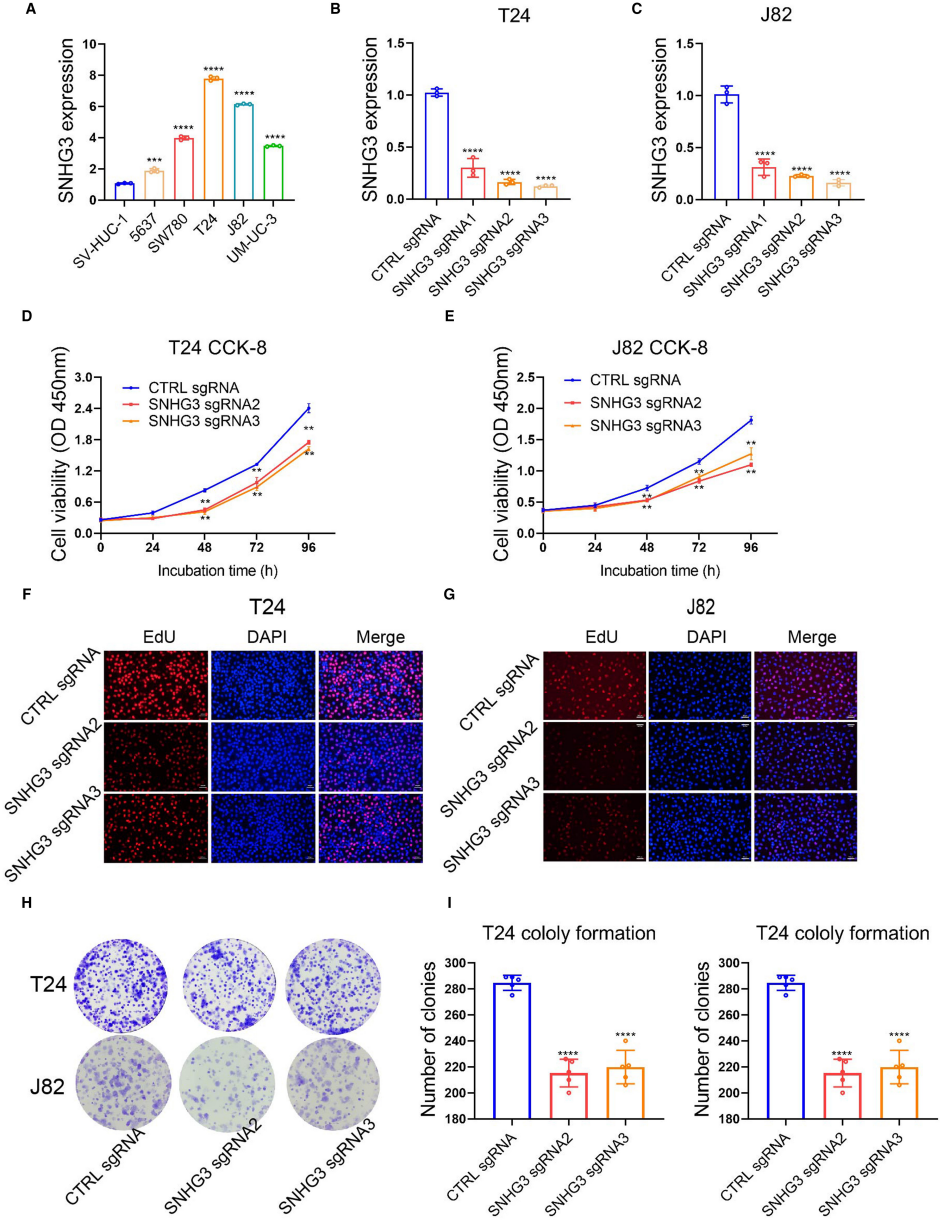
Knockdown of SNHG3 inhibits BLCa cell proliferation in vitro. (A) qRT‐PCR detection of SNHG3 expression levels in five BLCa cell lines (J82, 5637, T24, SW780, and UM‐UC‐3) and urothelial cell line SV‐HUC‐1 (control cells). (B, C) We constructed SNHG3 knockdown plasmids using CRISPR‐CAS9 technology and packaged them with lentiviruses, and then infected T24 and J82 with lentiviruses to construct BLCa cell lines that stably knocked down SNHG3. qRT‐PCR results showed that sgRNA2 and sgRNA3 had higher knockdown efficiency of SNHG3. The results of CCK8 assay (D, E), EdU staining (F, G) and plate colony formation assay (H, I) showed that knockdown of SNHG3 expression could significantly inhibit the proliferation of T24 and J82 cells. **p < 0.01, ***p < 0.001, ****p < 0.0001, ~versus CTRL sgRNA.

**FIGURE 3:**
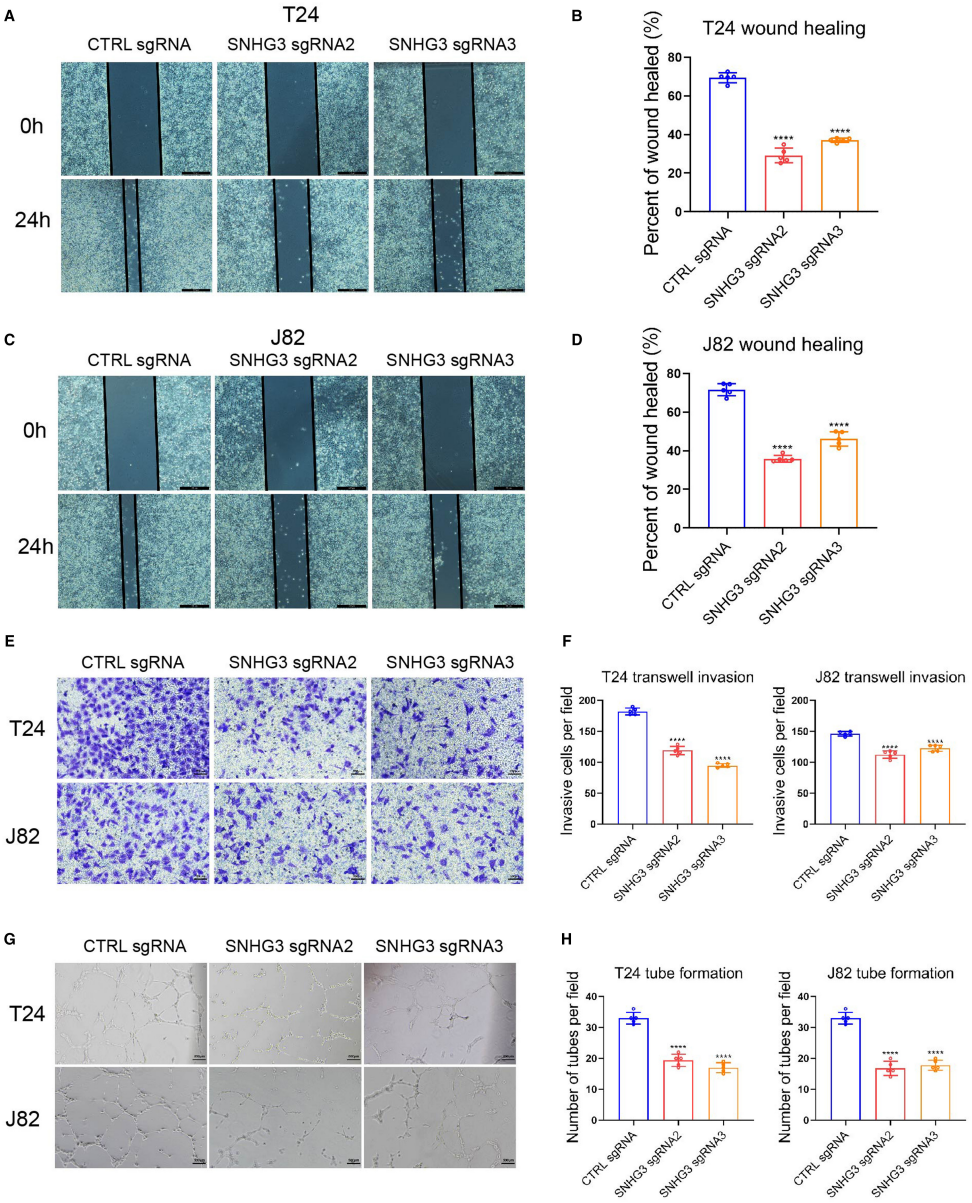
Knockdown of SNHG3 inhibits BLCa cell invasion, migration, and angiogenesis in vitro. The results of wound healing assay (A–D) and transwell invasion assay (E, F) showed that knockdown of SNHG3 expression could significantly inhibit the migration and invasion of T24 and J82 cells. (G, H) Vascular endothelial cell tube formation assay results showed that knockdown of SNHG3 expression could significantly attenuate the promoting effect of T24 and J82 cells on tubule formation. ****p < 0.0001, ~versus CTRL sgRNA.

First, we used qRT‐PCR to detect the expression levels of SNHG3 in five BLCa cell lines (J82, 5637, T24, SW780, and UM‐UC‐3) and a urothelial cell line SV‐HUC‐1 (control cells). Compared with SV‐HUC‐1, SNHG3 expression was higher in all five BLCa cell lines (Figure 2A). Then, we constructed SNHG3 knockdown plasmids using CRISPR‐CAS9 technology and packaged them with lentiviruses, and then infected T24 and J82 with lentiviruses to construct BLCa cell lines that stably knocked down SNHG3. The qRT‐PCR results showed that sgRNA2 and sgRNA3 had higher knockdown efficiency of SNHG3 **(**Figure 2B,C **)**. Subsequently, the results of CCK8 assay, EdU staining, and plate colony formation assays revealed that knockdown of SNHG3 could significantly inhibit the proliferation of T24 and J82 cells (Figure 2D–I). In addition, the results of both wound healing and transwell invasion assay suggested that knockdown of SNHG3 expression could significantly inhibit the migration and invasion of T24 and J82 cells (Figure 3A–F). Tumor angiogenesis is an important prerequisite for tumor invasion and metastasis. The results of vascular endothelial cell tube formation experiments demonstrated that knockdown of SNHG3 expression could significantly attenuate the promoting effect of T24 and J82 cells on tube formation (Figure 3G,H).

### 3.3 Overexpression of SNHG3 promotes BLCa cell proliferation, invasion, migration, and angiogenesis in vitro

**FIGURE 4:**
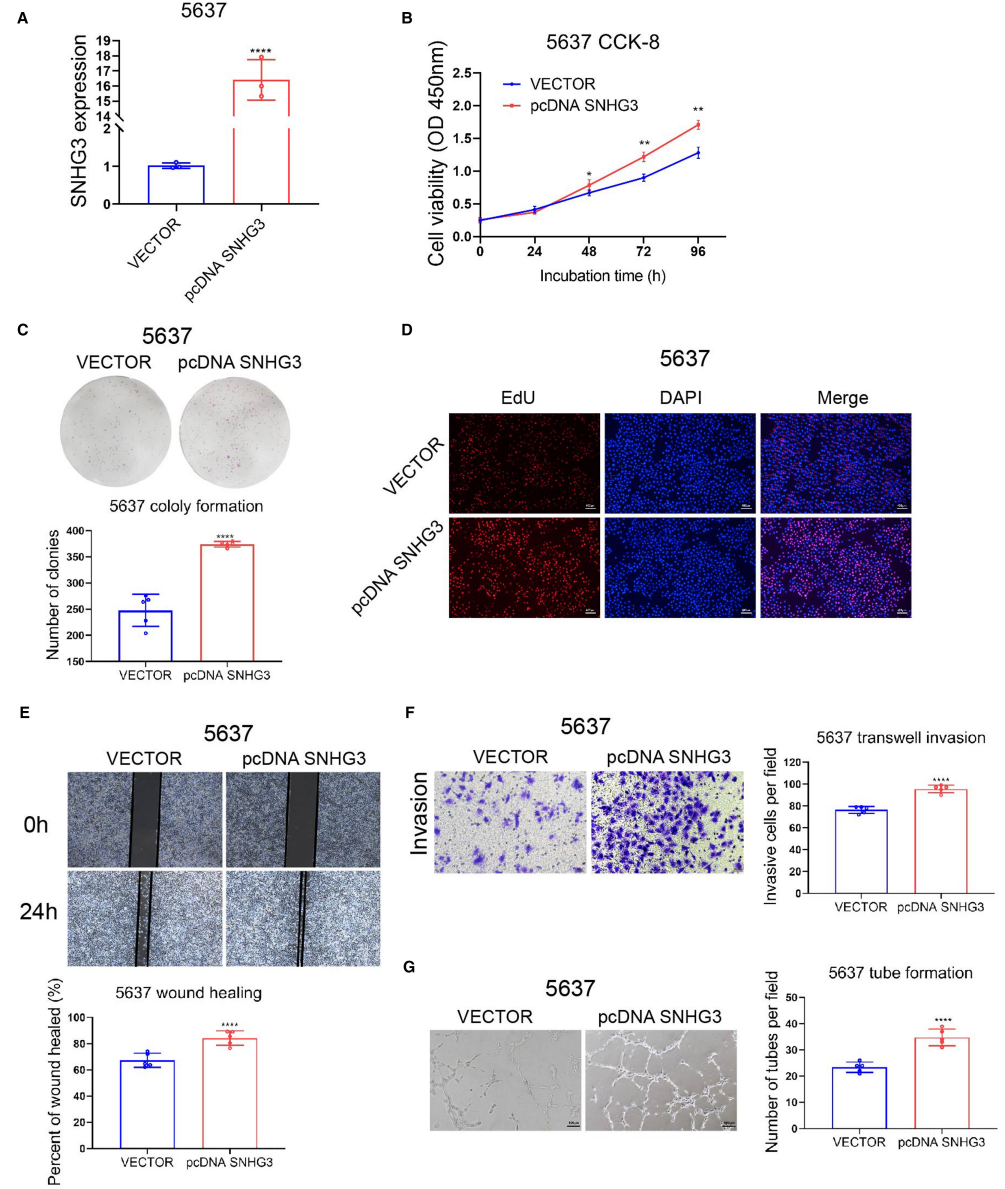
Overexpression of SNHG3 promotes BLCa cell proliferation, invasion, migration, and angiogenesis in vitro. (A) We transfected SNHG3 overexpression plasmid into 5637 cells and constructed stable cells; qRT‐PCR verified the overexpression efficiency. The results of CCK8 experiment (B), plate colony formation experiment (C), and EdU staining (D) showed that SNHG3 overexpression could significantly promote the proliferation of 5637 cells. The wound healing assay (E) and transwell invasion assay (F) showed that SNHG3 overexpression could significantly promote the migration and invasion of 5637 cells. (G) Vascular endothelial cell tubule formation experiments showed that overexpression of SNHG3 could enhance the promoting effect of 5637 cells on tubule formation. *p < 0.05, **p < 0.01, ****p < 0.0001, ~versus VECTOR.

First, we transfected SNHG3 overexpression plasmid into 5637 cells and constructed stable strains. The overexpression efficiency was verified by qRT‐PCR (Figure 4A). The results of CCK8 assay, EdU staining, and plate colony formation assays showed that SNHG3 overexpression could significantly promote the proliferation of 5637 cells (Figure 4B–D). The wound healing and transwell invasion assays suggested that SNHG3 overexpression could significantly promote the migration and invasion of 5637 cells (Figure 4E,F). In addition, the results of tube formation experiments in HUVECs demonstrated that overexpression of SNHG3 could significantly enhance promoting effect of 5637 cells on tube formation (Figure 4G).

### 3.4 LncRNA SNHG3 can bind c‐MYC protein in vitro

**FIGURE 5:**
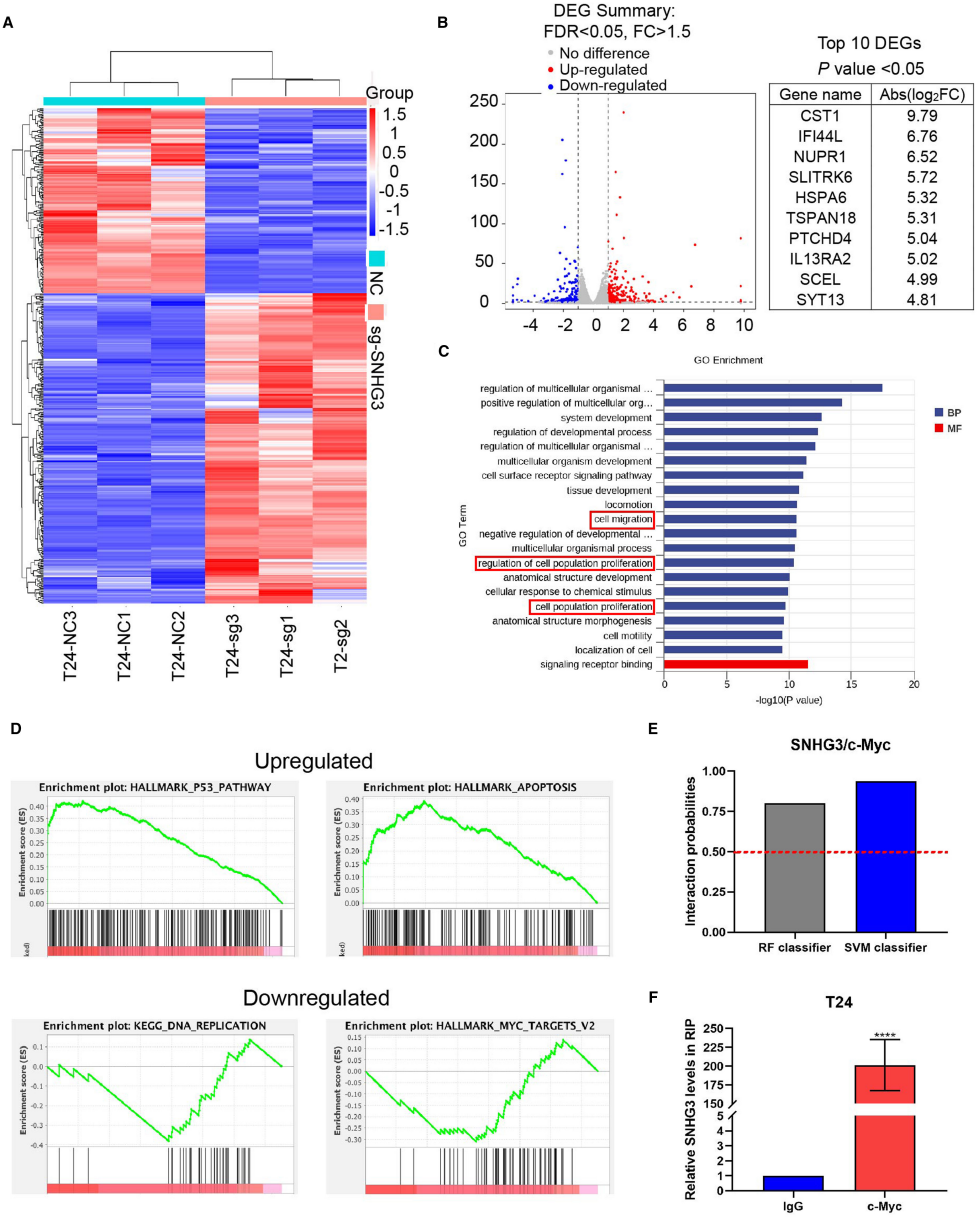
LncRNA SNHG3 can bind c‐MYC protein in vitro. (A, B) We performed RNA sequencing of SNHG3 knockdown T24 cells. Heatmap and volcano plot showed differentially expressed genes (DEGs). The top 10 differentially expressed genes were listed on the right panel of figure B. (C) Gene Ontology (GO) enrichment analysis indicated that knockdown of SNHG3 can regulate cell biological functions including cell migration and proliferation. We listed the top 20 potential biological functions. (D) GSEA enrichment were used to analyze the data and several representative hallmark pathways were exhibited. Hallmark gene signature in GSEA showed that SNHG3 can positively regulate the expression of the MYC family at the transcriptional level. (E) Online tool (http://pridb.gdcb.iastate.edu/ RPISeq/) predicted the binding of lncRNA SNHG3 to c‐MYC protein. When the probability of interaction>0.5, the target RNA and target protein can bind. (F) RIP and qRT‐PCR assays demonstrated that SNHG3 was included in the RNA co‐precipitated with c‐MYC protein. ****p < 0.0001, ~versus IgG control.

We performed RNA sequencing on SNHG3 knockdown T24 cells and analyzed the results. Heatmaps and volcano plots show differentially expressed genes (DEGs; Figure 5A,B). We listed the top 10 differentially expressed genes on the right panel of figure B (*p* < 0.05). Gene Ontology (GO) enrichment analysis indicated that knockdown of SNHG3 could modulate cellular biological functions including cell migration and proliferation (Figure 5C). Gene Set Enrichment Analyses (GSEA) were used to evaluate the Kyoto Encyclopedia of Genes and Genomes (KEGG) pathway and Hallmark gene signatures. The results showed that when SNHG3 was knocked down, the tumor suppressor‐related p53 pathway and apoptosis hallmark were significant upregulated, while DNA replication and hallmark MYC gene set were downregulated (Figure 5D). Then, we used an online bioinformatics tool (http://pridb.gdcb.iastate.edu/RPISeq/) to predict the binding of lncRNA SNHG3 to c‐MYC protein and the result showed that the probability of Interaction is over 0.5, which indicates the RNA may bind to target protein (Figure 5E). Subsequently, in vitro RNA Immunoprecipitation and qRT‐PCR demonstrated the inclusion of SNHG3 in RNA co‐precipitated with c‐MYC protein (Figure 5F).

### 3.5 Knockdown of SNHG3 may reduce the stability of BMI1 mRNA by inhibiting c‐MYC

**FIGURE 6:**
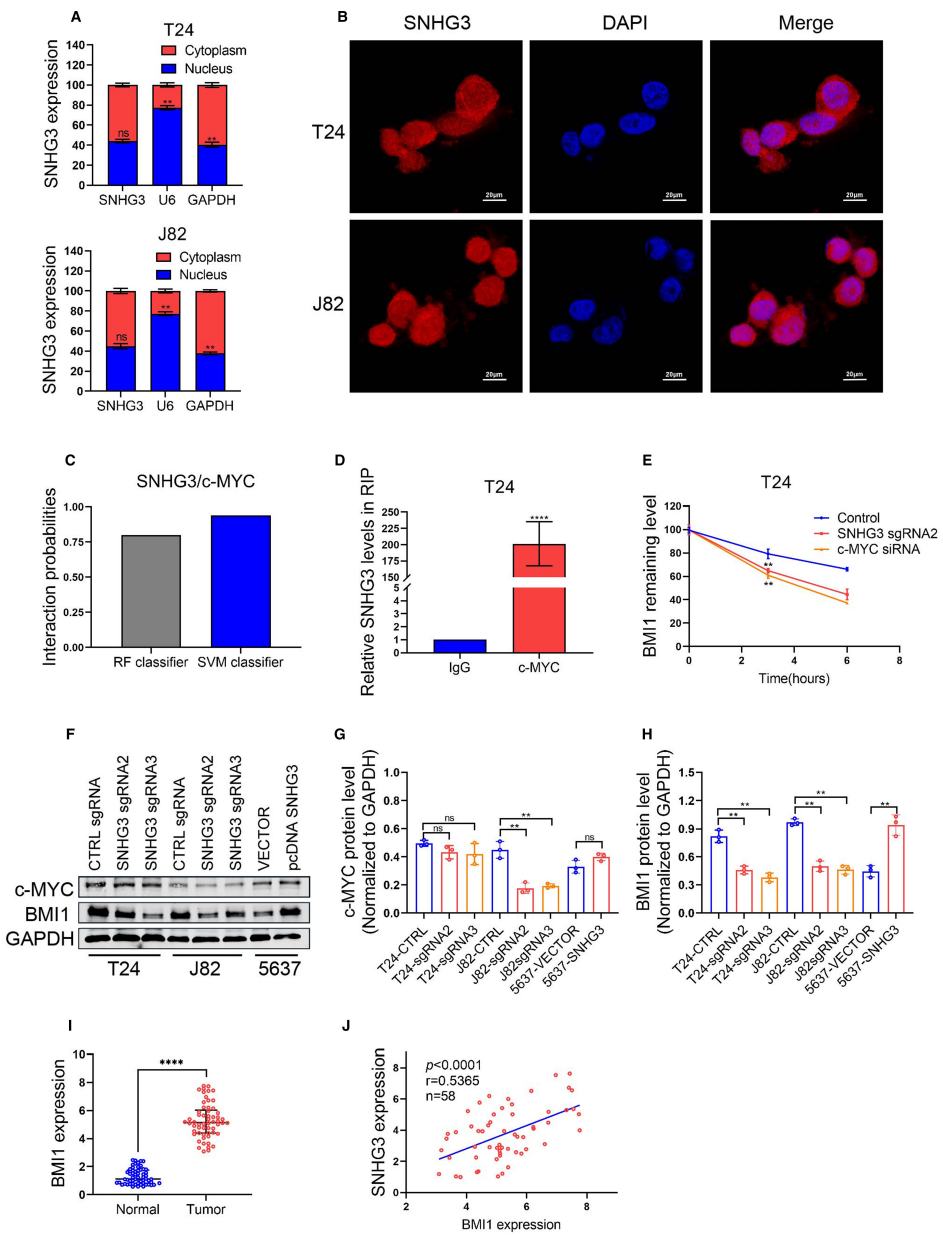
Knockdown of SNHG3 may reduce the stability of BMI1 mRNA by inhibiting c‐MYC. (A) We isolated cytoplasmic and nuclear RNA, and qRT‐PCR results showed that SNHG3 was slightly more abundant in the cytoplasm of T24 and J82 cells than in the nucleus (*p* > 0.05). U6 and GAPDH act as positive controls for the nuclear and cytoplasmic fractions, respectively. (B) FISH assay and confocal microscopy were used to investigate subcellular localizations of SNHG3 in T24 cells and J82 cells. (C) We used an online bioinformatics tool (http://pridb.gdcb.iastate.edu/RPISeq/) to predict the binding of BMI1 RNA to c‐MYC protein, and the result showed that the probability of Interaction is over 0.5, which indicates the target RNA may bind to target protein. (D) RIP and qRT‐PCR assays demonstrated that BMI1 was included in the RNA co‐precipitated with c‐MYC protein. ****p < 0.0001, ~versus IgG control. (E) RNA stability assay was performed using Actinomycin D to disrupt RNA synthesis in T24 cells, and degradation rates of BMI1 mRNAs were measured every 2 h. (F–H) Western blot showed that in three BLCa cells (T24, J82, and 5637), knockdown of SNHG3 could significantly inhibit the protein level of BMI1. However, surprisingly, knockdown of SNHG3 hardly affected the expression of c‐MYC, except in J82 cells, knockdown of SNHG3 slightly suppressed the expression of c‐MYC (*p* < 0.05). (I) BMI1 mRNA expression levels in 58 Pairs of BLCa tissues and adjacent normal tissues were detected by qRT‐PCR. (J) Correlation analyses showed that the expression levels of BMI1 and lncRNA SNHG3 were proportional in human BLCa tissues. Data are shown as mean ± standard errors. **p < 0.01, ****p < 0.0001.

First, we isolated the cytoplasmic and nuclear RNA of BLC cells, and the qRT‐PCR results showed that the content of SNHG3 in the cytoplasm of T24 and J82 was slightly higher than that in the nucleus, without no statistic difference (Figure 6A). According to FISH experiments and confocal microscopy observations, SNHG3 was evenly distributed in the cytoplasm and nucleus of T24 cells and J82 cells (Figure 6B). B lymphoma Mo‐MLV insertion region 1 (BMI1) has been shown to be an oncogene in various cancers including BLCa. Then, we used an online bioinformatics tool (http://pridb.gdcb.iastate.edu/RPISeq/) to predict the binding of BMI1 RNA to c‐MYC protein and the result showed that the probability of interaction is over 0.5, which indicates the target RNA may bind to target protein (Figure 6C). Subsequently, in vitro RNA immunoprecipitation and qRT‐PCR demonstrated the inclusion of BMI1 in RNA co‐precipitated with c‐MYC protein (Figure 6D). Then, we applied RNA stability assays to explore whether knockdown of SNHG3 or c‐MYC affected the stability of BMI1 mRNA. The results showed that knockdown of SNHG3 or c‐MYC could both reduce the stability of BMI1 mRNA compared with the negative control, but the inhibitory effect of knockdown of c‐MYC was more obvious (Figure 6E). Within here we hypothesized that SNHG3 may promote the stability of BMI1 mRNA by binding to c‐MYC and/or promoting the expression of c‐MYC. Western blot results showed that in three BLCa cells (T24, J82, and 5637), knockdown of SNHG3 could significantly inhibit the protein level of BMI1, while overexpression of SNHG3 could significantly increase the protein level of BMI1. However, surprisingly, knockdown of SNHG3 or overexpression of SNHG3 hardly affected the expression of c‐MYC, except in J82 cells, knockdown of SNHG3 could slightly inhibit the protein level of c‐MYC (Figure 6F–H), suggesting that knockdown SNHG3 may reduce the stability of BMI1 mRNA by inhibiting the protein level of c‐MYC, but this depends on the cell type. Finally, we found that BMI1 was highly expressed in BLCa tissues compared with adjacent tissues (Figure 6I) and was proportional to the expression level of lncRNA SNHG3 (*p* < 0.0001; Figure 6J).

### 3.6 Knockdown of BMI1 can significantly attenuate the tumor‐promoting effect of SNHG3 overexpression

**FIGURE 7:**
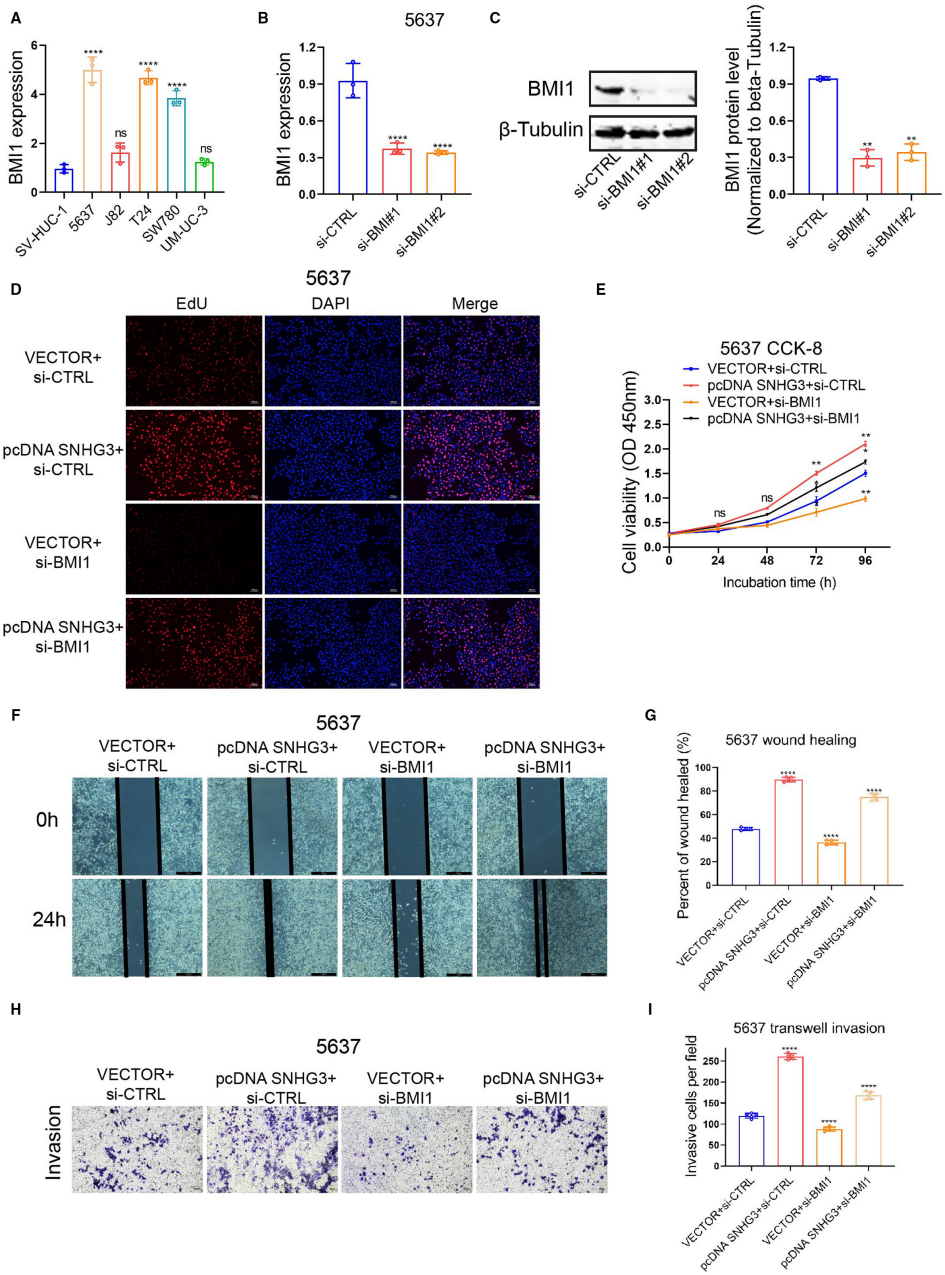
Knockdown of BMI1 significantly attenuates the tumor‐promoting effect of SNHG3 overexpression. (A) The expression levels of BMI1 in five BLCa cell lines (J82, 5637, T24, SW780, and UM‐UC‐3) and urothelial cell line SV‐HUC‐1 (control cells) were detected by qRT‐PCR. ****p < 0.0001, ~versus SV‐HUC‐1. (B, C) The expression of BMI1 was knocked down by siRNA interference technology, and the knockdown efficiency was verified by qRT‐PCR and Western blot. ****p < 0.0001, ~versus si‐CTRL. EdU staining (D) and CCK8 assay (E) results showed that knockdown of BMI1 could significantly inhibit the promoting effect of SNHG3 overexpression on the proliferation of 5637 cells. The results of wound healing (F, G) and transwell invasion assays (H, I) showed that knockdown of BMI1 could significantly inhibit the promotion of SNHG3 overexpression on the migration and invasion of 5637 cells. *p < 0.05, **p < 0.01, ****p < 0.0001.

We used qRT‐PCR to detect BMI1 expression levels in five BLCa cell lines (J82, 5637, T24, SW780, and UM‐UC‐3) and a urothelial cell line SV‐HUC‐1 (control cell). Compared with SV‐HUC‐1, the expression level of BMI1 was higher in all five BLCa cell lines (Figure 7A). Then, we knocked down the expression of BMI1 using siRNA interference technology and verified the knockdown efficiency using qRT‐PCR (Figure 7B) and Western blot (Figure 7C). The results of EdU staining and CCK‐8 assays showed that knockdown of BMI1 could significantly inhibit the promoting effect of SNHG3 overexpression on the proliferation of 5637 cells (Figure 7D,E). In addition, the results of wound healing experiments (Figure 7F,G) and transwell invasion experiments (Figure 7H,I) showed that knockdown of BMI1 could significantly inhibit the promotion of SNHG3 overexpression on the migration and invasion of 5637 cells.

### 3.7 Knockdown of SNHG3 significantly inhibits BLCa xenograft tumor growth

**FIGURE 8:**
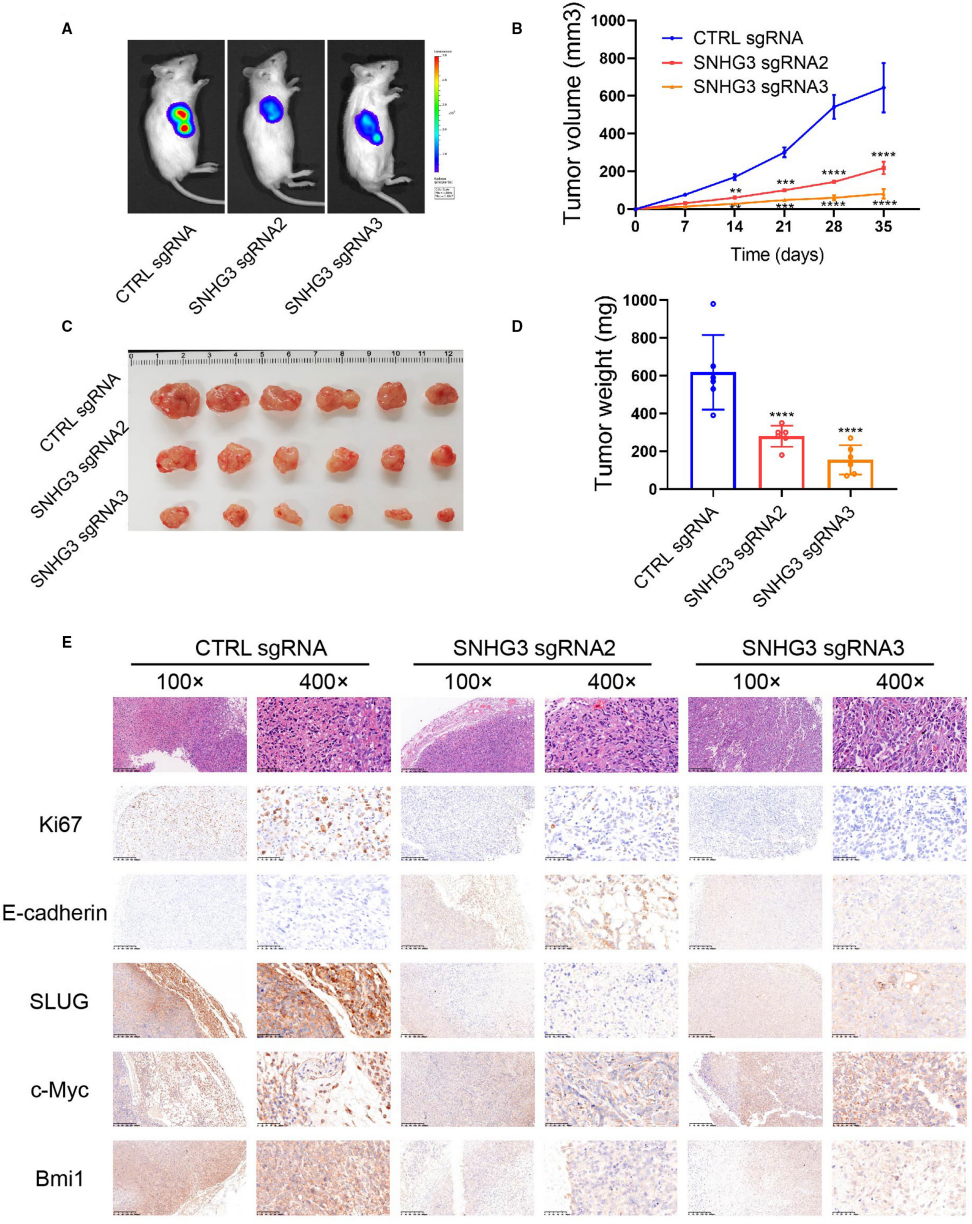
Knockdown of SNHG3 significantly inhibits BLCa xenograft tumor growth. (A) We infected T24 cells with sgRNA2 and sgRNA3 lentiviruses and constructed stable strains, and the empty vector (CTRL sgRNA) was used as a negative control. Subsequently, we inoculated the stable strains of each group into the armpits of M‐NSG mice. IVIS imaging was used to assess subcutaneous tumor growth, and representative images of mouse xenograft tumors were collected on the 28th day. (B) Tumor volumes were measured at different time points. (C) Representative images of gross xenograft tumors. (D) Tumor weights were measured after mice sacrifice. (E) H&E and IHC stainings were performed to detect the expression levels of Ki‐67, E‐cadherin, SLUG, c‐MYC, and BMI1 in tumors. **p < 0.01, ***p < 0.001, ****p < 0.0001, ~versus CTRL sgRNA.

First, we infected T24 cells with sgRNA2 and sgRNA3 lentiviruses and constructed stable strains with luciferase gene in vitro, and the empty vector (CTRL sgRNA) was used as a negative control. Subsequently, we inoculated the stable strains of each group into the armpits of M‐NSG mice. IVIS imaging was used to assess subcutaneous tumor growth on the 28th day (Figure 8A). In vivo results showed that the volume and weight of xenograft tumors derived from SNHG3 knockdown T24 cells were significantly smaller than those of the negative control **(**Figure 8B–D **)**. The results of H&E staining suggested that knockdown of SNHG3 could significantly inhibit the expression levels of Ki‐67 and BMI1 in xenograft tumors. However, knockdown of SNHG3 hardly affected the expression of c‐MYC. The above findings are consistent with the results of in vitro experiments. In addition, epithelial‐mesenchymal transition (EMT) is usually closely related to the occurrence and development of tumors. Immunohistochemical results demonstrated that knockdown of SNHG3 could promote the expression level of the epithelial marker E‐cadherin and inhibit the expression level of the mesenchymal marker SLUG **(**Figure 8E **)**.

## 4 DISCUSSION

Globally, BLCa is the ninth most common cancer.^24^ So far, surgery, chemotherapy, and radiotherapy are still the main therapeutics for BLCa. However, the 5‐year recurrence rate (15%–90%) and overall survival (50%–60%) of BLCa are still unsatisfactory.^25^ Therefore, researchers need to delve into the etiology of BLCa and identify better therapeutic targets.

The role of lncRNAs in the pathogenesis and progression of BLCa has attracted extensive attention and been reported continuously. Rui X et al.^26^ demonstrated that LinRNA GAS6‐AS2 promotes BLCa cell proliferation and metastasis through the miR‐298/CDK9 axis. Our group previously revealed that aberrant linc01451 in BLCa promotes EMT‐induced cancer progression by activating the TGF‐β/Smad signaling pathway.^27^ In addition, BLCa lymph node metastasis has also become of interest to researchers. He W et al.^28^ demonstrated that lincRNA BLACAT2 promotes VEGF‐C expression by binding to WDR5, a core subunit of the human H3K4 methyltransferase complex, which in turn promotes BLCa‐related lymphangiogenesis and lymph node metastasis. In‐depth study of the molecular regulatory mechanism of lncRNAs in BLCa will help to elucidate the high recurrence rate and rapid progression of BLCa, and also promote target‐oriented BLCa therapy.

The lncRNA SNHGs family is drawing increased interest. Our work and two other concurrent studies^21^, ^22^ confirmed that SNHG3 is a suitable target for the diagnosis and treatment of BLCa. Dai G et al.^21^ demonstrated that SNHG3 upregulates GINS2 expression by sponging miR‐515‐5p, which in turn promotes BLCa progression. However, in BLCa, SNHG3 may regulate cancer progression through different pathways. Mechanistically, our study found that SNHG3 can play a tumor‐promoting role by interacting with an intracellular target protein. Cao Y et al.^22^ demonstrated the role of SNHG3 in BLCa progression using CRISPR‐dCas9 technology. This study examined the effect of SNHG3 on several tumor phenotypes, and a series of in vitro functional experiments showed that SNHG3 overexpression could promote the proliferation, migration, invasion, and angiogenesis of BLCa cells, indicating SNHG3 was positively correlated with the malignant transformation of BLCa. However, they did not further explore the molecular regulatory mechanism of SNHG3. Compared with the study by Cao Y et al., we did validation work in vivo, and this will provide an experimental basis for subsequent studies on PK/PD and a safety evaluation of target molecules in animals or humans.

To explore the molecular mechanism of SNHG3's tumor‐promoting effect in BLCa, we performed RNA sequencing using T24 cells with SNHG3 knockdown. GSEA showed that SNHG3 could positively regulate the expression of the MYC gene set at the transcriptional level. MYC family proteins have been reported to promote tumorigenesis in several human malignances.^29^, ^30^, ^31^ Notably, c‐MYC is both an RNA‐ and DNA‐binding protein. Thus, we hypothesized that c‐MYC might act as a bridge in regulation of SNHG3 on downstream target genes. Zou Z et al.^32^ confirmed that LINC00324 stabilized the expression of FAM83B by binding to HuR, thereby promoting the proliferation of gastric cancer cells. Subsequently, we combined bioinformatics and in vitro experiments to demonstrate that lncRNA SNHG3 can bind c‐MYC protein in vitro. We hypothesized that SNHG3 regulates the expression of downstream target genes by binding to c‐MYC. Previously, studies have shown that the subcellular localization of lncRNA is critical for its functions.^6^ Several previous studies have shown that SNHG3 is widely distributed in the nucleus and cytoplasm of tumor cells.^33^, ^34^ We verified that SNHG3 was distributed both in cytoplasm and nucleus of BLCa cell by qRT‐PCR and FISH assays, which contradicts the findings of Dai G et al.^21^ They demonstrated that SNHG3 is mainly distributed in the cytoplasm. In BLCa, the exploration of the mechanism of interaction between LncRNA SNHG3 and RBP is innovative. The binding of SNHG3 to RBP can occur in both the nucleus and the cytoplasm, and our conclusions are also complementary to the ceRNA mechanism proposed by Dai G et al., because it is widely believed that ceRNAs are only applicable to ncRNAs in the cytoplasm.^6^, ^35^ Since the nucleus is the main site of gene transcription, we speculate that SNHG3 can regulate the transcription of target genes in nucleus.

It is well established that noncoding RNAs regulate downstream target genes at the transcriptional and post‐transcriptional levels. B lymphoma Mo‐MLV insertion region 1 (BMI1) has been shown to play an important role in various cancers and is strongly associated with poor prognosis.^36^, ^37^ Liang W et al.^38^ found that BMI1 was upregulated in BLCa tissues, and its knockdown could significantly impair the malignant phenotype of T24 cells. Our previous study found that MYC is closely associated with BMI1 in colorectal cancer.^39^ Another study has demonstrated that SNHG3 in liver cancer cells can regulate BMI1 through ceRNA mechanism, thereby affecting the malignant behavior of liver cancer.^37^ Within this work, we intended to verify whether SNHG3 could regulate BMI1 via an RBP. We combined bioinformatics and in vitro experiments to demonstrate that BMI1 mRNA can bind c‐MYC protein in vitro. RNA stability is an important factor affecting the intracellular abundance of RNA. Since c‐MYC can act as an RBP to stabilize mRNA in cancer,^40^, ^41^ we used actinomycin D to block the upstream transcription, then to evaluate the mRNA stability using qRT‐PCR assay. The results revealed that either SNHG3 interference or c‐MYC knockdown reduced the stability of BMI1 mRNA, but c‐MYC knockdown had a more significant inhibitory effect (Figure 6E). Therefore, SNHG3 can enhance the stability of BMI1 mRNA by binding to c‐MYC and/or promoting the expression of c‐MYC. To further verify our hypothesis, western blot assays demonstrated that SNHG3 knockdown significantly inhibited the protein level of BMI1 in three BLCa cell lines (T24, J82, and 5637). However, surprisingly, knockdown of SNHG3 hardly affected the c‐MYC expression, except in J82 cells, SNHG3 knockdown could slightly inhibit the c‐MYC expression (Figure 6F), suggesting that SNHG3 knockdown can reduce the stability of BMI1 mRNA by inhibiting the protein level of c‐MYC, but this depends on the cell type.

There are some limitations in this study. First, we need to confirm the co‐localization of SNHG3 and c‐MYC in the nucleus via combination of FISH and immunofluorescence assays. Second, we need to confirm whether the entry of c‐MYC into the nucleus is recruited by SNHG3 or autonomously. Third, in vitro functional assays demonstrated that knockdown of BMI1 could significantly attenuate the cancer‐promoting effect of SNHG3 overexpression, suggesting that the SNHG3/c‐MYC/BMI1 axis may play an important regulatory role in BLCa. However, we need to further explore the effect of SNHG3 knockdown on tumor metastasis in vivo. Our findings, combined with what has been reported, suggest that SNHG3 functions in BLCa through multiple parallel signaling axis. Future work needs to clarify whether their relationship is synergistic or antagonistic to explain some of the conflicting results. Taken together, our research is still in the early stages of new target development. There is still a lot of research work to carry out, such as half‐life, immunogenicity, safety, targeting, off‐target effects, PK, MOA, etc. In future, we will conduct planned research and provide more data to promote the clinical translation of SNHG3.

In conclusion, our study demonstrated that SNHG3 plays an oncogenic role in BLCa and is positively associated with poor prognosis in BLCa patients. In vitro, SNHG3 can stabilize BMI1 mRNA by binding to c‐MYC protein or regulating the expression of c‐MYC. SNHG3/c‐MYC/BMI1 axis may be a novel target for regulating tumor growth and metastasis in BLCa patients.

## AUTHOR CONTRIBUTIONS

**Jinbo Xie:** Conceptualization (equal); investigation (lead); project administration (equal); validation (equal); writing – original draft (lead). **Jinliang Ni:** Data curation (equal); investigation (equal); validation (supporting); writing – original draft (supporting). **Huajuan Shi:** Funding acquisition (supporting); resources (supporting); writing – review and editing (supporting). **Keyi Wang:** Data curation (supporting); visualization (supporting). **Xiaoying Ma:** Methodology (supporting); writing – review and editing (equal). **Wei Li:** Funding acquisition (supporting); visualization (supporting). **Bo Peng:** Conceptualization (lead); funding acquisition (lead); project administration (lead); resources (lead); supervision (lead); writing – review and editing (equal).

## FUNDING INFORMATION

This study was financially supported by the National Natural Science Foundation of China (Grant Nos. 31670772, 81870517, 81602469) and Shanghai Natural Science Foundation (No. 20ZR1443000), the Excellent Youth Foundation of Tongji University (No. 2016KJ045) and Foundation of Pujiang Talents Plan (No. 20PJ1412400), and Science and Technology Innovation Project of Putuo District System (ptkwws201916).

## CONFLICTS OF INTEREST

The authors declare that they have no competing interests.

## ETHICS STATEMENT

The approval ID for the use of animals was TJBA01920102 and is issued by Tongji University Laboratory Animal Welfare Ethics Review working Group Committee. The Clinical samples were collected with ethical approval from the Ethics Committee of Shanghai Tenth People's Hospital (SHSY‐IEC‐4.1/19–209/01).

## Supporting information


Appendix S1

## Data Availability

Data sharing is not applicable to this article as no new data were created or analyzed in this study.
