# Supplementary material for: LncRNA SNHG3 enhances BMI1 mRNA stability by binding and regulating c‐MYC: Implications for the carcinogenic role of SNHG3 in bladder cancer
Source: Cancer Med. 2022 Oct 8;12(5):5718–35. doi: 10.1002/cam4.5316 (PMC10028137; doi:10.1002/cam4.5316)
Supplement: Supplementary file 1 — Appendix S1 [file CAM4-12-5718-s001.docx]

**Supplemental Section 1：Materials and Methods**

**Cell culture**

The human BLCa cell lines (T24, 5637, SW780, J82 and UM-UC-3), immortalized normal human uroepithelium cell line (SV-HUC-1) and the human umbilical vein endothelial cell line (HUVEC) were purchased from the American Type Culture Collection (ATCC). T24, 5637, SW780, J82 and UM-UC-3 cells were cultured in RPMI-1640 medium (Gibco, USA) supplemented with 10% fetal bovine serum (FBS; Gibco; Thermo Fisher Scientific, Inc., USA), SV-HUC-1 cells were cultured in F12K medium (Gibco, USA) with 10% FBS, while HUVEC cells were cultured in Endothelial Cell Medium (iCell Bioscience Inc, Shanghai, China) supplemented with vascular endothelial growth factor (VEGF), insulin-like growth factor-1, basic fibroblast growth factor (bFGF), epidermal growth factor (EGF) and 2% FBS. All the above culture media were also supplemented with 100U/ml penicillin and 100 mg/ml streptomycin (Yeasen, Shanghai, China). Then, incubation was done in a humidified 5% CO_2_ atmosphere at 37˚C.

**RNA isolation and qRT-PCR analyses**

Total RNA was extracted using the TRIzol reagent (Invitrogen, Carlsbad, CA) as instructed by the manufacturer and stored in RNA-free H_2_O at -80℃. Nuclear and cytoplasmic RNA extraction was performed using Invitrogen kits (00738305, Thermo Fisher, USA) and protocols provided by the manufacturer. The qRT-PCT was performed on an ABI Prism 7500 sequence detection system (Applied Biosystems, USA) using the PrimeScript RT Reagent Kit and SYBR Premix Ex Taq (TaKaRa, Japan). The primers used in this assay (**Supplemental Section 7)** were designed and synthesized by Generay Biotech (Shanghai) Co., Ltd. Expression levels of RNA were calculated using the 2^-ΔΔCt^ method with Beta-actin (ACTB) as the internal reference.

**CCK-8 assay**

Each well of 96-well-plates was seeded with approximately 2000 BLCa cells in the logarithmic growth phase. At 0, 24, 48, 72, 96 h after seeding, each well was treated with 10 µl of the Cell Counting Kit-8 reagent (Yeasen, Shanghai, China) and incubated for 1 h at 37℃. Then, absorbance in each well was read at 450 nm using a microplate spectrophotometer (BioTek Instruments, Inc. USA).

**Colony formation assay**

Approximately 500 transfected cells were plated into 6-well plates and incubated for 2 weeks to form colonies. Then, colonies were counted after staining with crystalline purple.

**EdU assay**

The EdU assay kit was purchased from RiboBio (Guangzhou, China), and the assays were conducted according to the manufacturer's instructions. Briefly, transfected cells were seeded into a 24-well plate for 24 h. Then, cells were incubated with the EdU reagent for 4 h. Following washing, cells were fixed in paraformaldehyde for 30 min, decolorized using glycine for 5 min and permeabilized in 0.5% Triton X-100 for 10 min. Ultimately, cell images were obtained by fluorescence microscopy (Nikon, Japan) after being stained with the reaction solution.

**Wound healing assay**

Cells were seeded into a 6-well-plate to 90% confluence. Then, a 200 μl micropipette tip was used to scratch the confluent cell monolayers after which floating cells were scratched thrice and incubated in fresh medium for 24 h. At 0 and 24 h, images were captured using a Leica Microsystems microscope (Mannheim, Germany) and calculated using ImageJ software.

**Transwell assay**

To evaluate cell invasion abilities *in vitro*, transwell chambers (Corning, Inc., Lowell, MA, USA) and Matrigel were used. The upper chamber was filled with 200 μl of serum-free medium and inoculated with transfected cells (5 × 10^4^ cells/well) while 750 μl of medium containing 10% FBS was added to the bottom chamber. After 24 h of incubation at 37℃ in 5% CO_2_, cells attached to the upper chamber were carefully removeds, while cells located on the bottom chamber were fixed in 70% ethanol and stained with 0.1% crystal violet. Images were obtained by microscopy (Leica Microsystems, Mannheim, Germany).

**Western blot analysis**

Proteins were separated by sodium dodecyl sulfate-polyacrylamide gel electrophoresis (SDS-PAGE) after which 20 µg of protein samples were transferred to the Polyvinylidene Difluoride membrane (PVDF, Millipore, MA, USA). Membranes were blocked using 5 % skimmed milk for 1 h, incubated with primary antibodies at 4℃ overnight, washed 3 times using the TBST buffer, and incubated with secondary antibodies for 1 h at room temperature. Signals were visualized on the Odyssey Infrared Imaging System (LI-COR Biosciences, Lincoln, NE, USA). All the antibodies were as follows: anti-GAPDH (ab181602, Abcam, USA), anti-c-Myc (ab32072, Abcam, USA), anti-Bmi1 (ab126783, Abcam, USA) and goat anti Rabbit IRDye 800CW (926-32211, LI-COR, USA).

**Actinomycin D assay**

Transcription was interfered with by the addition of 2 μg/ml Actinomycin D (Sigma-Aldrich, USA) at the indicated time points. After treatment with Actinomycin D, RNA expression levels were analyzed by qRT-PCR.

**Supplemental Section 2：Genes from the intersection**

| Number | co_gene |
| --- | --- |
| 1 | ENO1 |
| 2 | MFAP2 |
| 3 | SNHG3 |
| 4 | GBP1P1 |
| 5 | MLLT11 |
| 6 | S100A11 |
| 7 | CCT3 |
| 8 | TMSB10 |
| 9 | HSPE1 |
| 10 | CXCL10 |
| 11 | SPP1 |
| 12 | TUBB |
| 13 | PDIA4 |
| 14 | LY6E |
| 15 | CKS2 |
| 16 | SNHG1 |
| 17 | PSMA4 |
| 18 | PSMD11 |
| 19 | SNORD104 |
| 20 | RPN2 |
| 21 | PSMD8 |
| 22 | MMP11 |
| 23 | MIF |
| 24 | MCM5 |

**Supplemental Section 3.1：The correlation between clinicopathological characteristics and SNHG3 expression level in 58 BLCa patients.**

| Characteristics | No. of patients (%) | SNHG3 expression  （Normalized to GAPDH） | *P* value*^*^* |
| --- | --- | --- | --- |
| Age |  |  |  |
| <65 years | 22 (37.9) | 3.50±1.80 | 0.303 |
| ≥65 years | 36 (62.1) | 3.97±1.62 |  |
| Gender |  |  |  |
| Female | 12 (20.7) | 3.47±1.81 | 0.494 |
| Male | 46 (79.3) | 3.85±1.68 |  |
| Differentiation (grade) |  |  |  |
| Well/Moderate (I/II) | 24 (41.4) | 3.14±1.57 | 0.016 |
| Poor (III) | 46 (58.6) | 4.22±1.66 |  |
| T stage |  |  |  |
| T1 (NMIBC) | 32 (55.2) | 2.52±0.93 | <0.0001 |
| ≥T2 (MIBC) | 38 (44.8) | 5.21±1.14 |  |
| TNM stage |  |  |  |
| I/II | 47(81.0) | 3.43±1.62 | 0.001 |
| III/IV | 11 (19.0) | 5.26±1.16 |  |

TNM, tumor-node-metastasis; NMIBC, non-muscular invasive bladder cancer; MIBC, muscular invasive bladder cancer.

* Independent-sample t-test.

**Supplemental Section 3.2：Details of the patients**

| **ID** | **Gender** | **Age** | **Year of diagnosis** | | **Grade** | | **TNM**  **stage** | **T stage** | **Survival months** | **Status** |
| --- | --- | --- | --- | --- | --- | --- | --- | --- | --- | --- |
| P1 | Male | 68 | 2015 | I | | I | | T1 | 14 | Dead |
| P2 | Female | 76 | 2011 | I | | I | | T1 | 48 | Alive |
| P3 | Male | 58 | 2011 | III | | I | | T1 | 49 | Alive |
| P4 | Male | 59 | 2011 | II | | I | | T1 | 8 | Dead |
| P5 | Female | 63 | 2011 | III | | I | | T1 | 39 | Alive |
| P6 | Female | 64 | 2011 | II | | I | | T1 | 48 | Alive |
| P7 | Male | 59 | 2011 | III | | I | | T1 | 51 | Alive |
| P8 | Female | 78 | 2011 | III | | I | | T1 | 50 | Alive |
| P10 | Male | 72 | 2011 | I | | I | | T1 | 39 | Alive |
| P11 | Male | 68 | 2012 | III | | I | | T1 | 43 | Dead |
| P12 | Male | 53 | 2015 | II | | I | | T1 | 5 | Alive |
| P13 | Male | 63 | 2012 | II | | I | | T1 | 39 | Alive |
| P14 | Male | 79 | 2014 | II | | I | | T1 | 9 | Dead |
| P15 | Male | 73 | 2012 | I | | I | | T1 | 52 | Alive |
| P16 | Male | 55 | 2012 | II | | I | | T1 | 49 | Dead |
| P17 | Male | 75 | 2012 | III | | I | | T1 | 15 | Alive |
| P18 | Male | 84 | 2012 | II | | I | | T1 | 70 | Alive |
| P19 | Male | 58 | 2012 | III | | I | | T1 | 59 | Alive |
| P20 | Male | 63 | 2012 | II | | I | | T1 | 39 | Alive |
| P21 | Female | 62 | 2014 | II | | I | | T1 | 11 | Alive |
| P22 | Male | 62 | 2013 | I | | I | | T1 | 50 | Dead |
| P23 | Male | 76 | 2013 | II | | I | | T1 | 31 | Alive |
| P24 | Male | 83 | 2014 | II | | I | | T1 | 49 | Alive |
| P25 | Male | 52 | 2014 | II | | I | | T1 | 58 | Alive |
| P26 | Male | 74 | 2011 | III | | I | | T1 | 55 | Alive |
| P27 | Male | 71 | 2015 | III | | IV | | T1 | 41 | Alive |
| P28 | Male | 67 | 2011 | III | | II | | T2a | 40 | Alive |
| P29 | Female | 73 | 2011 | III | | II | | T2a | 51 | Alive |
| P30 | Male | 52 | 2011 | III | | III | | T4a | 36 | Alive |
| P30 | Male | 84 | 2013 | III | | I | | T1 | 62 | Dead |
| P31 | Male | 72 | 2011 | III | | II | | T2a | 70 | Alive |
| P32 | Female | 83 | 2015 | III | | I | | T1 | 32 | Dead |
| P33 | Male | 72 | 2011 | II | | I | | T1 | 29 | Dead |
| P34 | Male | 57 | 2015 | III | | I | | T1 | 19 | Alive |
| P35 | Male | 80 | 2011 | III | | I | | T1 | 14 | Dead |
| P36 | Male | 73 | 2011 | III | | II | | T2a | 68 | Alive |
| P37 | Male | 85 | 2012 | II | | II | | T2a | 9 | Dead |
| P38 | Male | 64 | 2012 | III | | II | | T2a | 52 | Alive |
| P39 | Male | 62 | 2012 | III | | II | | T2a | 52 | Alive |
| P40 | Female | 61 | 2012 | III | | IV | | T2b | 40 | Dead |
| P41 | Male | 67 | 2013 | II | | II | | T2a | 2 | Alive |
| P42 | Male | 71 | 2011 | III | | II | | T2a | 60 | Dead |
| P43 | Female | 60 | 2014 | II | | II | | T2a | 2 | Alive |
| P44 | Male | 60 | 2012 | III | | IV | | T3a | 7 | Alive |
| P45 | Male | 62 | 2012 | III | | III | | T3a | 14 | Dead |
| P46 | Female | 81 | 2012 | III | | III | | T3b | 1 | Alive |
| P47 | Male | 71 | 2012 | III | | III | | T4a | 36 | Dead |
| P48 | Female | 81 | 2015 | II | | II | | T2a | 37 | Dead |
| P49 | Male | 66 | 2015 | III | | II | | T2a | 57 | Alive |
| P50 | Male | 71 | 2013 | III | | III | | T3a | 49 | Dead |
| P51 | Male | 71 | 2015 | III | | II | | T2a | 19 | Dead |
| P52 | Female | 70 | 2012 | III | | IV | | T4a | 8 | Alive |
| P53 | Male | 54 | 2012 | III | | IV | | T3b | 11 | Dead |
| P54 | Male | 84 | 2013 | III | | II | | T2a | 3 | Dead |
| P55 | Male | 52 | 2012 | II | | II | | T2a | 8 | Alive |
| P56 | Male | 81 | 2012 | II | | II | | T2b | 38 | Alive |
| P57 | Male | 60 | 2012 | III | | II | | T2b | 64 | Alive |
| P58 | Male | 82 | 2012 | III | | III | | T4a | 17 | Dead |

**Supplemental Section 4：SNHG3 small nucleolar RNA host gene 3 [ *Homo sapiens* (human) ]**

Gene ID: 8420, updated on 21-Jun-2020

NR_002909.2

gattctctaactgcgcatgcttctgcgcacgcgcaatagacattccaggacttccgggcacttcgtaaggtttaaaaaggatgcttcgcgttttctctctcctttttggagacagattcgcagtggtcgcttcttctccttggatttgttaaggattccaagtaactcttatttggagagaagacgatctgcacttcgcattttggcattgacatttaattttagggtcctttatatagaagggagagtaggtaaactgatttttttttttaacagggagggtttgacaatctttggcagacttggagcaaaagattgaggtgcatttcatgcctccttttgagagtcttgctctgtcgcccaggctgtagtgcagtggcgcaatcttggctgcaacctcagcctcccaagtagctgggattacaaacataagccaccacgcccagccctcatacctcttttaaaagtcgacctgttttgcagaaagtctgctgtttttgtactaaaggctttggaatttggcatttagctaggaatgcacattctttcacctcattcatactttaagaaccacagaagtgactctgcttggccagaaggcacactgtgttggtggttatattaaaagtccttgagtattttgcttttcatgatcttgctcactgcaacttccgcctcccaggttcaggcgattctcctgcctcagcctcccaagtagctgcgactacaggcgtgtagcaccacacctggctaatttttgtatttttagtagagatgaggtttcaccatattggccaggctgttctcaactcctgacctcgtgatccgcccacctcagcctcctaaagtgctgggattacagctgtgagccaccctgcccggccacttttgtatgatttctaatgtatttgtaatttacctaacaaattgcctaatctgctatgttaatgtatttatgaattaaaataaatacgactgcatgtttgtggttcatttttgtggaggtggctgtggtgacatcagccaagaatctgaatggtactgttgaaggaaactagcatgatagcttcagttctaaaggccctgaaacctagtctcaggtgggtcccccttgggttcactttatattggcagtttattgggaaaatggatattaggtcctgaccaataggaccgtaagtctgggttgagtgcaagatgagttagaccgattctttagcttcctgcagtgtagtggaggaaaaatcgatggtagcaacgggaggttgtatccctagctgatgagttgtatgagcctctactacctggcgcacctccgcctgaagattgccagaattgcttgcctcatgacgtgagtcacaatggaaactttgtcaagccccctgcactggctgccaacataaatgttcagtaccctgaaggatgggactgaagggggatcatctagaaggtaaagttacctactggcataggggaggtgggacagccgttaagccatttggaacttgatggagacaggtttgagggaggtgggtgagattggagtttggtggactgtagagcttgcttgccaaggtgttgaggtcagggttggtttgagaatggaagctagttactagctatgattgtgggggaacacagcttgatttttcttacaagctaagaggagtgaggcagtgtttaagagggcatgttaaatgcagccaggcttggtggctcacacccgtaatcccagcacttaggctaaggcaggcggatcacaacatctagagatcctggccaacgcggtgaaaccctgtctgtactaaaaatacaaaataactgggcatggtggtgtgcacctgtgggaggctgaggcagaattgctggaacccgggagatggaggttgtactgagctgagaccttgccactgcgctccagcctggtgacagagttaagtctcaaaaaaaaggcatcttcctaaagcaattgtatttgtgcttacctgtgccaggcactgttctaggtaagcactaagtgggctttaatacagcatattccaatggggaatcccaggaaccaaaagactaattgtccaagtccacaactagaagtggcacctctgcagaaacaagcatcaaattccctgctcaggaagaagccagatgagtcagccccattcgtctgtatgcccagtcccatccgtgtcctgctgtaactacatagatctcacctgagtaaagtgatttttttctgaa

**Supplemental Section 5：sgRNA Target design**

sgRNA1：GGACTTCCGGGCACTTCGTA F


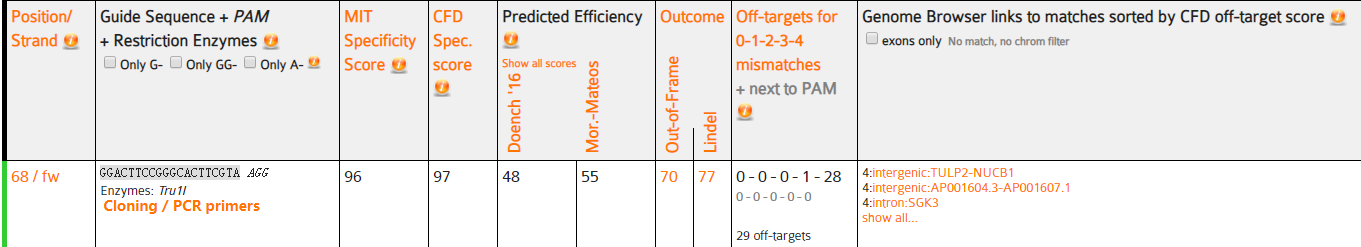


sgRNA2：GACTTACGGTCCTATTGGTC R


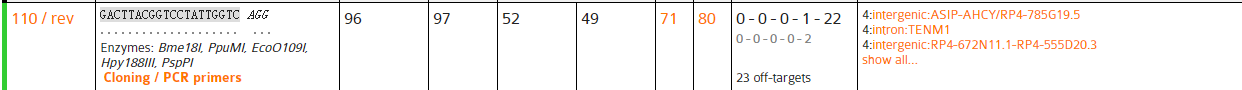


sgRNA3：ACTGGGCATACAGACGAATG R


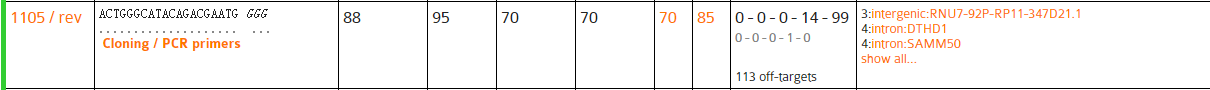


**Supplemental Section 6：The primer sequences**

| Primer | Sequences (5’ to 3’) |
| --- | --- |
| ACTB-F | CATGTACGTTGCTATCCAGGC |
| ACTB-R | CTCCTTAATGTCACGCACGAT |
| SNHG3-F | CTCCCAAGTAGCTGCGACTA |
| SNHG3-R | CAGCCTGGCCAATATGGTGAA |
| Bmi1-F | GTCACCAGAGAGATGGACTGA |
| Bmi1-R | GAAGAGGTGGAGGGAATACCT |
